# Serotype diversity of *Actinobacillus pleuropneumoniae* detected by real-time PCR in clinical and subclinical samples from Spanish pig farms during 2017–2022

**DOI:** 10.1186/s13567-024-01419-2

**Published:** 2024-12-18

**Authors:** José Luis Arnal Bernal, Marcelo Gottschalk, Sonia Lacotoure, Celia Sanz Tejero, Gema Chacón Pérez, Desiree Martín-Jurado, Ana Belén Fernández Ros

**Affiliations:** 1 Exopol. Pol, Río Gállego D14, San Mateo de Gállego, Zaragoza Spain; 2 Research Group on Infectious Diseases in Production Animals and Swine and Poultry Infectious Diseases Research Centre, Faculty of Veterinary Medicine, University of Montreal, 3200 Sicotte, Saint-Hyacinthe, QC J2S 2M2 Canada

**Keywords:** *Actinobacillus pleuropneumoniae*, serotype, real-time PCR

## Abstract

*Actinobacillus pleuropneumoniae* is the causative agent of porcine pleuropneumonia, a challenging respiratory disease for the global swine industry. Variations in the serotypes associated with clinical disease have been observed in different regions worldwide. This study aimed to provide an updated epidemiological assessment of *A. pleuropneumoniae* serotypes in Spain, incorporating bacterial characterization through serotyping and toxinotyping. Serotypes 9/11, 2, 4, 5, 17, and 13 were frequently identified in diseased animals. Furthermore, qPCR of lung samples from an outbreak, even when samples were pooled, emerged as a robust diagnostic tool, enabling the rapid detection of *A. pleuropneumoniae* and their serotypes without the need for microbiological isolation. This technology also facilitates serotype monitoring of apparently healthy herds through the testing of oral fluids. The study revealed the frequent simultaneous presence of diverse serotypes within a farm. Serotypes 1, 7, 10, 12, 18, and 19 were frequently found in subclinically infected animals but were rarely detected in acute pleuropneumonia outbreaks in the current study. These results provide valuable information for interpreting the potential virulence of the different serotypes in Spain. However, other predisposing factors and the immune status of the herds such as type of vaccines used when appropriate, should be carefully considered before drawing definitive conclusions. Nevertheless, the study offers valuable insights that underscore the necessity for detailed regional data to contribute toward a comprehensive understanding of the disease dynamics and toward formulating effective control measures for porcine pleuropneumonia.

**Supplementary Information:**

The online version contains supplementary material available at 10.1186/s13567-024-01419-2.

## Introduction

*Actinobacillus pleuropneumoniae* is the aetiological agent of porcine pleuropneumonia. It is part of the porcine respiratory disease complex (PRDC) and is spread worldwide [1]. This illness mostly affects fattening and adult animals and is of major concern in pig production because of the significant economic losses that it causes [2]. Not only the mortality associated with acute outbreaks but also the increased costs related to antimicrobials and vaccination should be taken into account. Furthermore, *A. pleuropneumoniae* frequently persists as a subclinical infection, with apparently healthy animals serving as reservoirs harbouring the pathogen within their tonsillar tissues and subsequently shedding it. Some strains may persist in a herd for extended periods of time without any clinical signs exhibited by the animals, or any chronic lesions detectable at the abattoir [1]. Nevertheless, the insidious progression of the disease clearly undermines production metrics, as reflected by the diminished rates of daily weight gain and feed efficiency, with potential decline of up to 25% [3, 4].

*Actinobacillus pleuropneumoniae* is a highly diverse bacterial species. The study of its diversity has been approached through various methods. The requirement of nicotinamide adenine dinucleotide (NAD) for microbiological growth defines biotype I (NAD-dependent) and biotype II (NAD-independent) strains [5]. Moreover, differences in the antigenicity of the capsular polysaccharides determine the serotype. A total of 19 serotypes have been described to date [6, 7], although serotypes 9 and 11 cannot be easily differentiated and are normally detected together [8, 9]. It is worth emphasizing that immunity resulting from infection is directed against the corresponding serotypes [1]. Regardless of serotype, all virulent strains possess the necessary genes to produce and secrete at least one of the tree main virulence factors, the pore forming exotoxins ApxI, ApxII and ApxIII [10, 11]. Each serotype typically exhibits a consistent pattern of these toxins, generally leading to significant differences in virulence [12]. However, there are exceptions; despite sharing similar toxin profiles, certain serotypes (e.g. 7 and 12) display considerable differences in virulence [1]. Furthermore, notable exceptions within the same serotype have been documented, which limit the discriminatory capacity of the Apx system [13, 14]. Therefore, serotyping remains the most widely accepted method for characterizing *A. pleuropneumoniae* and is crucial for implementing effective control measures.

Serotyping of *A. pleuropneumoniae* was originally carried out with antisera. However, the homology of common epitopes between certain serotypes limits the accuracy of the identification and generates diverse cross-reactions [1]. Molecular methods such as polymerase chain reaction (PCR) [8] or real-time quantitative PCR (qPCR) [15] appeared to solve this issue and allowed laboratories to avoid misidentifications. Nonetheless, these molecular serotyping tools still need microbiological isolation. A previous study [16] developed a method for the identifying and typing *A. pleuropneumoniae* in lung homogenates from clinically affected animals. The limited sensitivity of conventional PCR [7] would most likely restrict its application to animals in the acute phase of the disease, where a high bacterial load is expected to be recovered. Implementing a sensitive and reliable method for the detection and serotyping of *A. pleuropneumoniae* both from clinical samples and from carrier animals would greatly enhance diagnostic and monitoring processes.

Important differences in the serotype distribution of isolates involved in clinical outbreaks have been described worldwide [15, 17–21]. Furthermore, the epidemiological situation within a specific geographical area can change after a period of time [22–26]. Spain is currently the leading swine producer in Europe and the third worldwide [27]. However, the available epidemiological data are scarce and needs to be updated. The data from a study [28] published a decade ago are likely no longer representative of the current situation. There is only one recent study [29] conducted in this country. It is based on whole genome sequencing techniques and suggests that the most prevalent serotypes are 2, 4, 9/11, 13, and 17.

Only one serotype is usually recovered from each clinical outbreak [1]; nevertheless, it is known that several serotypes can be subclinically present in a farm and even in a single animal [30]. There are still gaps in our knowledge of which factors trigger a specific serotype from a subclinical infection to cause an acute outbreak. It is accepted that there are other PRDC pathogens such as *Mycoplasma hyopneumoniae* [31]*,* swine influenza virus (SIV) [32], and porcine circovirus type 2 (PCV2) [33] whose concomitant infection enhances the potential virulence of *A. pleuropneumoniae*, while the role of others, like porcine reproductive and respiratory syndrome virus (PRRSV) [34] is still under discussion. There is great interest in the ability to predict the virulence of a specific serotype once it has entered a farm. Consequently, it would be very valuable to obtain up-to-date, local data on the serotypes most frequently associated with outbreaks as well as on those present but not linked to significant issues.

Current molecular techniques represent cost-effective diagnostic tool that yields unambiguous results. The use of qPCR enables the detection of certain parameters directly from clinical samples such as from lungs or oral fluids [35], thereby overcoming some of the shortcomings of microbiological culture.

Considering all the above, our objective was to present comprehensive epidemiological data on the distribution of *A. pleuropneumoniae* in Spain. Through an updated survey, we aimed to provide information on the serotypes involved in clinical outbreaks as well as to identify those present in subclinically infected animals using innovative molecular techniques such as qPCR. To complete the epidemiological study and assess the variability of this agent in Spain, we also investigated the intrinsic characterization of the bacteria through serotyping, biotyping, and virulotyping of a representative collection of isolates.

## Materials and methods

The study used material submitted from Spanish commercial pig farms to the Exopol laboratory (Zaragoza, Spain) for diagnostic services during the period 2017–2022. A collection of *A. pleuropneumoniae* isolates obtained from a selection of clinical cases were examined. Additionally, two distinct types of samples that tested positive for *A. pleuropneumoniae* by qPCR were included. Lung samples were obtained from diseased animals exhibiting evident respiratory signs and categorized as clinical cases. Oral fluids were collected from farms without any ongoing respiratory disorders, where the analysis was aimed at herd monitoring, and these were designated as subclinical cases.

### Biological material studied

#### *A. pleuropneumoniae* isolates

The serotype of 262 *A. pleuropneumoniae* isolates obtained from the lungs of sick animals at necropsy was determined by qPCR. These isolates originated from 240 distinct outbreaks, encompassing at least 188 different farms distributed across 23 Spanish provinces. The study of each outbreak included three to five animals. Typically, a single isolate per outbreak was selected, except in cases where both biotypes were microbiologically detected, hence resulting in a higher number of isolates than outbreaks. Lung tissue was cultured on Columbia agar supplemented with 5% defibrinated sheep blood (Oxoid Ltd., Basingstoke, Hampshire, UK). Immediately after being plated, a *Staphylococcus aureus* streak was made in the plate and incubated at 37 °C overnight. Growth colonies were identified by MALDI-TOF MS using a Microflex LT/SH system with the Biotyper database (Bruker Daltonics, Bremen, Germany) and subcultured in chocolate agar (Oxoid Ltd.). Biotype identification was conducted by growing the bacteria on Columbia sheep blood agar (Oxoid Ltd.) at the same time as *S. aureus* streaking to provide the NAD factor. Isolates that grew only near the streak were considered biotype I, while those that were able to grow independently of the distance to the streak were classified as biotype II.

Among the 262 isolates, 78 representing the most relevant serotypes were selected for analysis of the genes coding for toxins ApxI, ApxII, and ApxIII. The inclusion criteria for the toxin genes study were serotype detection frequencies greater than 5%. Additionally, and when available, isolates from different biotypes within the same serotype were included. Furthermore, two isolates of serotype 1 were also selected because of their known virulence in other geographic regions and their novel detection in Spain. The collection included isolates from the following serotypes: serotype 1 (*n* = 12), serotype 2 biotype I (*n* = 14), serotype 2 biotype II (*n* = 16), serotype 4 (*n* = 16), serotype 5 (*n* = 10), serotype 9/11 (*n* = 13), serotype 13 (*n* = 22), serotype 17 biotype I (*n* = 12), and serotype 17 biotype II (*n* = 1).

#### Samples from clinical cases

The study included samples from 712 respiratory clinical cases collected throughout the aforementioned period: 2017 (*n* = 6), 2018 (*n* = 81), 2019 (*n* = 226), 2020 (*n* = 188), 2021 (*n* = 96), and 2022 (*n* = 115). Each case was represented by one unique tissue sample from one animal (*n* = 344 cases) or a pool of up to five samples from different animals simultaneously selected from the given outbreak. The distribution of cases with pools of different size was: 2 animals (*n* = 223 cases), 3 animals (*n* = 91 cases), 4 animals (*n* = 36 cases), and 5 animals (*n* = 18 cases). An additional study (see Additional file 1) confirmed the reliability of pooling up to five lung samples without loss of sensitivity. This collection originated from at least 474 different farms across 33 Spanish provinces. However, for 63 samples, the farm of origin was not provided. The discrepancy between the number of clinical cases and the number of farms is due to the fact that certain fattening farms submitted samples from outbreaks occurring in different years, where the origin of the animals may not necessarily be the same. The lung tissue samples were included in the study if they met two criteria: they were taken from animals showing signs of respiratory disease and they tested positive for *A. pleuropneumoniae* species via qPCR with a cycle of quantification (Cq) value of ≤ 30. This cut-off was established based on a previous study that compared the sensitivity (Se), specificity (Sp), positive predictive value (PPV) and negative predictive value (NPV) using various cut-off Cq values (38, 32, 30, 28, 26, 24, 22 and 20). For this purpose, a collection of 834 lung tissue samples from animals with respiratory disease was analysed for the presence of *A. pleuropneumoniae* via qPCR and microbiological isolation, with the latter traditionally regarded as the gold standard diagnostic method.

#### Samples from subclinical cases (carrier animals)

Additionally, 172 oral fluid samples that had previously tested positive for *A. pleuropneumoniae* by qPCR were included in this study. This material was sampled from animals with no obvious signs of respiratory disease, with their vaccination status remaining undetermined. Each oral fluid sample was considered an aggregate sample since several animals contribute to its formation. These samples were obtained from animals in the post-weaning or fattening stages, originating from at least 50 farms across 17 Spanish provinces. Although detailed pen size information for each sample was unavailable, it is important to note that, according to the requirements of the Spanish production system, the maximum number of animals per pen is usually 30 in the post-weaning stage and 15 in the fattening stage. Inclusion criteria mandated solely the presence of *A. pleuropneumoniae*, as confirmed by qPCR, irrespective of the Cq value.

### Molecular detection

All the isolates, tissues, and oral fluids were analysed using the qPCR technique. First, nucleic acids were extracted and purified using the MagMAX CORE Nucleic Acid Purification Kit (Applied Biosystems, Austin, TX, USA) following the manufacturer’s instructions with the KingFisher Flex device (Thermo Scientific, Rockford, IL, USA). Tissue specimens (lungs, joints, brain) were pre-treated using the MagMAX CORE Mechanical Lysis Module (Applied Biosystems, Austin, TX, USA) through two runs of 6000 rpm for 30 s in a MagNA Lyser (Roche Diagnostics, Penzberg, Germany). The elution volume was modified from the user manual to reach 200 µL of elution buffer in order to provide enough volume for all the qPCR reactions required.

Once DNA was purified, a complete set of qPCR assays was performed for *A. pleuropneumoniae* species detection and respective serotyping. The commercially available qPCR kit EXOone *Actinobacillus pleuropneumoniae* (ref. APPL) (Exopol, Zaragoza, Spain) was used for *A. pleuropneumoniae* detection in the range of samples studied. This kit provides quantitative information on *A. pleuropneumoniae* through the 6-carboxyfluorescein (6-FAM) channel targeting the omlA gene. Moreover, the quality control of the molecular detection process was verified in all samples except isolates through the amplification of an endogenous control (hexachloro-fluorescein, HEX channel). Afterwards, all the *A. pleuropneumoniae* serotypes described to date were studied using the commercial kits EXOone *A. pleuropneumoniae* serotype 1–19 (ref. AP01-AP19) (Exopol, Zaragoza, Spain). These kits facilitate the specific detection of the 19 serotypes targeting the cps genes. The qPCR assays were validated using a collection of *A. pleuropneumoniae* reference strains (serotypes 1–19) kindly provided by the University of Montreal. The validation data are presented in the Additional file 2. The qPCR reactions were performed in a QuantStudio 5 Real-Time PCR System (Applied Biosystems, Austin, TX, USA) under the conditions specified by each manufacturer. The results were analysed using QuantStudio software v1.5.2. All results with a cycle threshold of ≤ 38 were considered positive.

The toxinotyping (Apx-typing) was performed via detection of the respective coding genes following a previously described method [36]. Since the reference strains of serotypes 1 and 2 contain *ApxI* + *ApxII* and *ApxII* + *ApxIII*, respectively, a mixture of their extracted nucleic acids (1:1) was used as positive control.

### Interpretation of results and statistical analysis

Isolates and tissue samples from clinical outbreaks that tested negative for the 19 serotypes studied were classified as non-typeable (NT). Differences in the frequency of detection of each serotype in isolates and lung tissues were compared. Fisher’s exact test was employed to identify differences between groups (*p < 0.05, **p < 0.01, and ***p < 0.001) using R Studio software, V4.0.3. The frequency of detection for each serotype was calculated over the entire study period (2017–2022) as well as annually. The evolution of the most prevalent serotypes, defined as those with a rate of detection of >5%, was assessed over time. Finally, the frequency of detection of each serotype from the 171 subclinical samples (oral fluids) was observed and compared with that in lung tissues from outbreaks. The number of subclinical samples in which a different number of serotypes was detected was recorded. Oral fluids testing negative for any serotype were considered NT.

## Results

The results supporting the cut-off of Cq < 30 for classifying a sample within the clinical case category are presented in Table 1. *A. pleuropneumoniae* was isolated in 197 out of 834 samples. The Cq < 30 cut-off achieved the highest sensitivity and specificity values, both exceeding 94%; it was therefore chosen as the criterion for classifying lung samples as part of the clinical cases of respiratory disease under investigation.

**Table 1:** **Assessment of Cq value cut-off for defining** ***A. pleuropneumoniae*** **qPCR-positive samples as clinical cases**

Cq value cut-off	Se	CI 95%		Sp	CI 95%		PPV	NPV
38	98.5	0.95	1.00	78.6	0.75	0.82	58.79	99.4
32	94.9	0.91	0.97	92.5	0.90	0.94	79.57	98.33
30	94.4	0.90	0.97	94	0.92	0.96	83.04	98.2
28	92.4	0.88	0.96	95	0.93	0.96	85.05	97.58
26	89.8	0.84	0.93	96.1	0.94	0.97	87.62	96.84
24	88.8	0.83	0.93	95.9	0.94	0.97	87.06	96.52
22	74.1	0.67	0.80	97.3	0.96	0.98	89.57	92.4
20	43.1	0.36	0.50	98.4	0.97	0.99	89.47	84.84

Se: sensitivity, Sp: specificity, PPV: positive predictive value, NPV: negative predictive value.

The frequency of detection for each serotype in isolates and lungs is presented in Table 2. There were no statistically significant differences (*p* < 0.05) within any serotype according to the results of Fisher’s exact test.

**Table 2:** **Frequency of detection of each serotype found in both isolates and clinical samples**

Serotype	Isolates (*n* = 262)			Lungs (*n* = 712)		
	Isolates	Percentage (%)	CI 95%	Samples	Percentage (%)	CI 95%
1	2	0.8	0.09–2.73	4	0.6	0.15–1.43
2^a^	50^e^	19.1	14.51–24.3	157	22	19.06–25.28
3	1	0.4	0.01–2.11	1	0.1	0–0.78
4	34	13	9.16–17.66	80	11.2	9.01–13.79
5	27	10.3	6.90–14.64	56	7.9	6.0–10.09
6	4	1.5	0.42–3.86	11	1.5	0.77–2.75
7	7	2.4	1.08–5.43	15	2.1	1.18–3.45
8	9	3.4	1.58–6.42	26	3.6	2.40–5.31
9/11	73	27.9	22.52–33.71	200	28.1	24.81–31.55
10	3	1.1	0.24–3.31	3	0.4	0.09–1.23
12	3	1.1	0.24–3.31	5	0.7	0.23–1.63
13^b^	23	8.8	5.65–12.88	90	12.6	10.29–15.31
14	0	0	0–1.40	0	0	0–0.52
15	1	0.4	0.01–2.11	0	0	0–0.52
16	0	0	0–1.40	0	0	0–0.52
17^c^	24	9.2	5.96–13.32	84	11.8	9.52–14.40
18	1	0.4	0.01–2.11	13	1.8	0.98–3.10
19	0	0	0–1.40	0	0	0–0.52
NT^d^	0	0	0–1.40	2	0.3	0.03–1.01

The percentages in clinical samples add up to more than 100% since some samples contained more than one serotype. No statistical differences (Fisher exact test; *p* < 0.05) were found when comparing the frequency of detection of each serotype from isolates and lungs.

^a^Serotype 2 isolates biotype I (*n* = 37, 75.5%) and biotype II (*n* = 12, 24.5%).

^b^All the serotype 13 isolates resulted in biotype II.

^c^Serotype 17 isolates included biotype I (*n* = 23, 95.8%) and biotype II (*n* = 1, 4.2%).

^d^Non-typeable.

^e^One isolate was initially determined as negative for serotype 2 by qPCR; however, subsequent whole-genome sequencing identified it as serotype 2.

### *A. pleuropneumoniae* isolates

In the analysis of the *A. pleuropneumoniae* isolates from our collection (*n* = 262), all serotypes except 14, 16, and 19 were detected. Serotype 9/11 was the most commonly found (27.9%, *n* = 73), followed by serotype 2 (19.1%, *n* = 50), 4 (13%, *n* = 34), 5 (10.3%, *n* = 27), 17 (9.2%, *n* = 24), and 13 (8.8%, *n* = 23). The rest of the serotypes were occasionally detected, with their collective rate of detection reaching 11.8%. All isolates but one yielded a positive result for a single serotype. The remaining isolate did not yield positive results in any of the serotyping qPCR assays; nonetheless, further investigations based on whole-genome sequencing revealed this to be serotype 2. Its cps gene contains two insertion elements (ISApl1), which hindered its identification by the current qPCR method. Considering the requirement for the NAD factor for growth, 226 isolates (86.2%) were confirmed as biotype I, and 36 (13.7%) as biotype II. Biotype II isolates accounted for all of the isolates of serotype 13, for 12 isolates of serotype 2 (24.5%), and for one isolate of serotype 17 (4.2%). All the isolates belonging to the rest of the serotypes were confirmed to be biotype I. The results of the Apx-typing of the selected isolates are shown in Table 3. The serotype 2 isolates exhibited variations in the Apx coding gene pattern depending on their biotype. All serotype 2 biotype I isolates, except one, harboured ApxII coding genes exclusively (*IBD*, *IICA*). By contrast, all serotype 2 biotype II isolates also possessed ApxIII coding genes (*IIICA* and *IIIBD*). Conversely, no differences in the Apx coding gene pattern were observed among the biotypes of serotype 17 isolates. Interestingly, one isolate from serotype 4 lacked ApxIII coding genes (*IIIBD* and *IIICA*). However, apart from these exceptions, all other strains within the same serotype and biotype displayed identical Apx coding gene patterns.

**Table 3:** **Biotype and toxinotype results from selected isolates belonging to the most prevalent serotypes**

Serotype	Biotype	n	Detected genes (Sthitmatee) [33]	ApxI	ApxII	ApxIII
1	I	2	*IBD*, *ICA*, *IICA*	x	x	
2	I	13	*IBD*, *IICA*, *IIICA*, *IIIBD*		x	x
2	I	1	*IBD*, *IICA*		x	
2	II	16	*IBD*, *IICA*		x	
4	I	15	*IBD*, *IICA*, *IIICA*, *IIIBD*		x	x
4	I	1	*IBD*, *IICA*		x	
5	I	10	*ICA*, *IBD*, *IICA*	x	x	
9/11	I	13	*ICA*, *IBD*, *IICA*	x	x	
13	II	22	*IBD*, *IICA*		x	
17	I	12	*IBD*, *IICA*		x	
17	II	1	*IBD*, *IICA*		x	

### Clinical cases

In the analysis of lung tissue samples (*n* = 712), all serotypes except 14, 15, 16, and 19 were detected. Serotype 9/11 was the one most often identified (28.1%, *n* = 200) followed by serotype 2 (22.0%, *n* = 157), 13 (12.6%, *n* = 90), 17 (11.8%, *n* = 84), 4 (11.2%, *n* = 80), and 5 (7.9%, *n* = 56). The remaining serotypes were hardly detected, and their collective abundance was 11.8%. Two samples (0.3%) did not yield positive results for any serotype and were considered NT. Overall, 95.1% (*n* = 677) of the samples had a single serotype, whereas two or three serotypes were observed in 4.5% (*n* = 32) and 0.1% (*n* = 1) of the samples, respectively (Table 4). Among the samples in which multiple serotypes were detected, 71.28% (*n* = 28) consisted of pooled lungs. There were only six samples of a single lung, representing 0.8% of the entire collection of tissue samples, with two different serotypes detected.

**Table 4:** **Number of different serotypes found in clinical and subclinical samples**

Number of different serotypes	Diseased animals		Carrier animals	
	Lungs	(%)	Oral fluids	(%)
0	2	0.28	2	1.17
1	677	95.08	67	39.18
2	32	4.49	31	18.13
3	1	0.14	25	14.62
> 3			46	28.89
Total	712		171	

**Figure 1:**
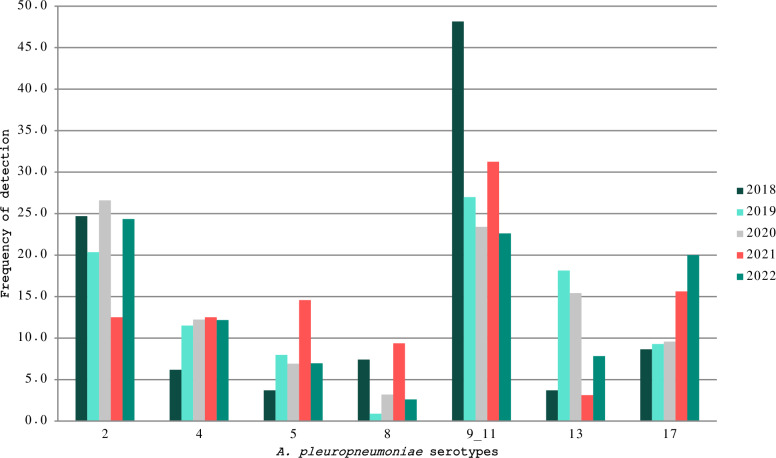
**Evolution of detection rates of the most frequently detected** ***A. pleuropneumoniae*** **serotypes during 2018–2022.**

The evolution of the detection rates of the most frequently detected serotypes (2, 4, 5, 8, 9/11, 13, and 17) during the study period is illustrated in Figure 1. The ranking of serotypes in terms of frequency of detection remained consistently unchanged. Serotypes 9/11 or 2 consistently emerged as the most prevalent throughout the study period from 2018 to 2022. The limited number of cases studied in 2017 precluded their inclusion in this estimation. Moreover, different trends were observed in some serotypes. Serotype 9/11 showed a decreasing occurrence, ranging from 48.1% in 2018 to 22.6% in 2022 (*p* < 0.001). For serotype 17, an increasing pattern was found, ranging from 8.6% in 2018 to 20% in 2022 (*p* < 0.05). The detection rate of serotype 4 ranged from 6.2 to 12.5%, but statistically significant differences were not observed. The detection of serotype 8 and 13 was inconsistent and ranged from 2.6 to 9.4% and from 3.1 to 18%, respectively.

### Subclinical cases

All serotypes except serotype 16 were detected when the samples from carrier animals were analysed. The *A. pleuropneumoniae* qPCR Cq value ranged from 22.38 to 37.9. The frequency of detection of each serotype is presented in Table 5. Serotype 7 was the most frequently found (42.7%), followed by serotypes 2 (33.9%), 12 (24.5%), 4 (23.9%), and 1, 9/11, and 17 (16.9%). Overall, 1.1% of the samples (*n* = 2) did not yield positive results for any of the serotypes and were considered NT. Their Cq values for *A. pleuropneumoniae* were 34.4 and 35.9, respectively.

**Table 5:** **Frequencies of detection of** ***A. pleuropneumoniae*** **serotypes found in clinical and subclincal animals**

Serotype	Lungs (*n* = 712)			Oral fluids (*n* = 171)		
	*n*	Percentage (%)	CI 95%	*n*	%	CI 95%
1	4	0.6	0.15–1.43	29	16.96	11.66–23.44
2	157	22	19.06–25.28	58	33.92	26.87–41.54
3	1	0.1	0–0.78	6	3.51	1.30–7.48
4	80	11.2	9.01–13.79	41	23.98	17.79–31.09
5	56	7.9	6.0–10.09	9	5.26	2.43–9.76
6	11	1.5	0.77–2.75	10	5.85	2.84–10.49
7	15	2.1	1.18–3.45	73	42.69	35.17–50.47
8	26	3.6	2.40–5.31	19	11.11	6.82–16.81
9/11	200	28.1	24.81–31.55	29	16.96	11.66–23.44
10	3	0.4	0.09–1.23	12	7.02	3.68–11.94
12	5	0.7	0.23–1.63	42	24.56	18.31–31.72
13	90	12.6	10.29–15.31	39	22.81	16.75–29.83
14	0	0	0–0.52	19	11.11	6.82–16.81
15	0	0	0–0.52	1	0.58	0.01–3.21
16	0	0	0–0.52	0	0	0–2.13
17	84	11.8	9.52–14.40	29	16.96	11.66–23.44
18	13	1.8	0.98–3.10	21	12.28	7.77–18.16
19	0	0	0–0.52	16	9.36	5.44–14.75
NT^a^	2	0.3	0.03–1.01	2	1.16	0.14–4.16

^a^Non-typeable.

Subclinical samples contained a variable number of different serotypes (Table 3). Most frequently, only one serotype was found (*n* = 67, 39.2%) followed by two serotypes (*n* = 31, 18.1%) and three serotypes (*n* = 25, 14.6%). In 28.9% of the cases (*n* = 46), more than three serotypes were detected.

## Discussion

This study provides updated data on the epidemiology of *A. pleuropneumoniae* in Spain. Although some surveys have been published in other European countries in recent years, this type of information has been lacking for Spain. One of the most innovative features of our work is that the detection of *A. pleuropneumoniae* serotypes was made not only from isolates but also directly from the clinical samples without the need for microbiological isolation. This represents a major difference from the most recent reports in the literature. Studies from Germany in 2022 [15] and the United Kingdom in 2016 [37] used multiplex conventional PCR to characterize the microbiological isolates. A survey from Hungary in 2018 [38] still used the indirect agglutination (IHA) serological test in which microbiological culture was also necessary. Moreover, although there is already a description of a high-resolution melting assay (qPCR) for *A. pleuropneumoniae* serotyping [15], its use without the prerequisite of microbiological isolation has not been reported.

The method used in this study for detecting *A. pleuropneumoniae* serotypes in clinical lung samples was based on several key aspects. First, the commercial qPCR assays were validated and corroborated using a collection of reference strains (Additional file 2). Additionally, it was demonstrated that pooling up to five lungs from animals within the same outbreak does not result in a loss of sensitivity (Additional file 1). Finally, the sensitivity (Se) and specificity (Sp) values, compared with the reference microbiological technique, support the use of a cut-off (Cq < 30) as the criterion for categorizing animals affected by pleuropneumonia (Table 1).

According to the data presented in Table 2, there were no statistical differences were observed in the detection rates of each serotype when isolates and lungs were compared. Although the results of the isolates and lungs did not stem from the same outbreaks, the large size of both sample collections—262 and 712, respectively—along with the high agreement in the results allows us to propose direct detection in lungs as a valid method for *A. pleuropneumoniae* serotyping when diagnosing clinical pleuropneumonia. Our findings validate this novel serotyping approach, which will facilitate faster and direct diagnosis in the future.

The diversity of serotypes identified in this study is extensive. All serotypes, except for 14, 16, and 19, were detected in clinical cases, with only two instances in which lung samples lacked characterization and were classified as non-typeable (NT). There was a particularly higher presence of serotype 9/11 in Spain (28%) compared with Germany in 2021 (15%) [18] and Hungary in 2018 (9%) [38]. Additionally, serotype 2 also demonstrated a considerable spread, albeit with rates significantly lower than those reported in Germany (64%) and Hungary (34%). Within this serotype we found the majority of biotype I strains (75.5%, *n* = 37) and a minor presence of biotype II strains (24.5%, *n* = 12), with each biotype presenting its own Apx toxin coding gene profile. All serotype 2 biotype I isolates but one presented *IBD*, *IICA*, *IIICA* and *IIIBD* genes, whereas the serotype 2 biotype II isolates carried *IBD*, *IICA* genes. These figures agree with those reported in the study from Germany [18], where a few serotype 2 biotype II isolates also presented only ApxII coding genes (*IBD*, *IICA*). Serotypes 9/11 and 2 were responsible for 50% of the clinical cases under study. To a smaller extent, serotypes 4, 5, 13, and 17 were also detected, with rates of around 10%. Serotype 4 seems to remain delimited to Spain [28], since there is no report of this serotype outside the country. The presence of serotype 5 was also reported in previous studies from Europe [18, 38], Australia [23], North America [24], and the Philippines [19]. Analysis of serotype 13 identified in this study consistently resulted in biotype II, similar to findings described in other European countries [15, 18, 38], suggesting a probable connection with the strain isolated in Denmark at the end of the 1990s [39]. The widespread presence of Danish genetic lines in major European intensive swine production companies could explain the dissemination of this serotype in recent years. The European serotype 13 isolates differ from contrast with the isolate found in North America, which belonged to biotype I [14]. Furthermore, the recently described serotype 17 is considerably spread throughout Spanish herds. To date there are few data on the presence of this serotype in Europe, and our work is the first to report on serotype 17 extensively. Notably, isolates from both biotypes were identified within this serotype. Indeed, the epidemiology of *A. pleuropneumoniae* around the world is highly diverse and changes continuously. Serotype 8 is scarcely found (3%) in Spain, while it is predominant in England and Wales [17, 37]. Serotype 7 is very frequently detected in Canada [24] and Australia [23], whereas we found only 2% of clinical cases related to this serotype. Our study delineates, for the first time, certain serotypes within the European context. Two isolates were identified as serotype 1. Although this serotype was of major concern in North America [24], it has not been reported in Europe to date. Serotype 15 was also found once; this serotype had been detected only in Australia [21] and Brazil [25].

This study compares data from a considerably long period: 2018–2022 (Figure 1). Since all the material was collected by a commercial laboratory and no sample design was implemented, these data do not provide a proper prevalence rate but instead they indicate a trend. Serotype 9/11 seems to have a decreasing trend, whereas serotype 17 displays a rising pattern. There were statistically significant differences for both serotypes between the years 2018 and 2022. These results should be considered with caution. Important differences were observed within each series; therefore, it is important to include several years in the analysis to minimize bias and to provide a more realistic view of the epidemiological situation.

Prior to this study, few data were available on the epidemiology of porcine pleuropneumonia in Spain. The most recent work [28] was published over a decade ago. This period represents a long time for an intensive swine-producing country like Spain where the livestock population has been constantly modified through the import of animals with high genetic value as well as piglets for fattening.

Moreover, the development of molecular methods has replaced the use of limiting serological methods such as coagglutination. These factors may account for several of the differences between our study and the Spanish study published by Maldonado et al. [28]. Biotype II isolates currently comprise 13.7% (*n* = 36) of cases, considerably less (25%) than the formerly observed rate. Moreover, the most frequently detected serotypes in both studies varied. On the one hand, the rate for serotype 4 was over 40% in the Maldonado et al. study, while we obtained a rate of barely 10% in the current study. Serotype 9/11 was of little importance 10 years ago; however, it is the most frequently detected serotype today. On the other hand, the figures for serotypes 2 and 5 have remained constant. Serotype 17, which was first described after the publication by Maldonado et al. [28], currently shows a significant presence.

The results regarding the Apx coding gene profiles were as expected for each of the respective serotypes [36]. Only two isolates, one from serotype 2 biotype I and another from serotype 4, showed an atypical pattern lacking ApxIII coding genes (*IIICA* and *IIIBD*). The former, although rare in Europe, is common in North America [1]. Other examples of unusual toxinotypes have been widely described before [13, 21, 40]. Since Apx toxins are considered to be the main virulence factor of this bacteria, it is worth highlighting that Spanish isolates from serotype 2 biotype II as well as serotype 13 and 17 only presented ApxII coding genes (*IBD* and *IICA*). These serotypes represent one fourth of the isolates detected in cases of clinical pleuropneumonia in the country. Therefore, according to our experience, those isolates coding only for one Apx toxin cannot be confirmed as being of low virulence and should not be neglected.

The utility of this study for the investigation of carrier animals constitutes a significant area of interest. The presence of several serotypes was demonstrated in healthy animals. The fact of a single herd [38] or even and individual animal [30] harbouring multiple serotypes has been previously documented. However, the number of different serotypes found simultaneously in oral fluids taken from healthy animals is much higher than that found in lungs and in previously published results for tonsils. These results should be interpreted by considering that the oral fluid provides in vivo information about the bacterial content of the tonsils from several animals simultaneously. Since the tonsils play the role of a reservoir throughout the life of the pig, analysing their content is not considered an acute diagnostic method but a monitoring tool. Previous studies of subclinical animals used earlier serological techniques, which had several shortcomings including cross-reactivity and limited sensitivity due to the lack of active infection. The use of new molecular methods with oral fluids enabled the direct detection of the selected genes when the bacteria are present. Consequently, this has demonstrated frequent infections by concurrent serotypes within a herd [35].

Swine pleuropneumonia is a multifactorial disease and the intrinsic characteristics of the *A. pleuropneumoniae* strain are not always sufficient to cause the disease. Although there is great interest in predicting which serotypes present in the herd are potentially more virulent and hence will trigger the disease, the available data are still limited. Thus, the valuable observational information offered by this work could be used to draw some conclusions, which, nevertheless, should be regarded with caution, in view of the limitations in the temporal and geographical scope of the data.

All serotypes but 16 were detected in oral fluids from apparently healthy animals. Interestingly, some serotypes such as 14 and 19, which were not observed in diseased animals, actually appeared in the oral fluids. At this point, it is important to underscore the almost complete homology exhibited in the *cps* genes which serve as the targets for the qPCR assays used in serotyping, between strains of serotype 14 and *Actinobacillus suis* [41]. This resemblance hinders the unequivocal confirmation of serotype 14 detection in oral fluids without excluding the potential presence of *A. suis*. Although serotype 14 has been reported in Hungary [38], it has not been identified in any other European country, including Spain. Therefore, data on this particular serotype should be interpreted with caution, as the results may actually indicate the presence of *A. suis*. Moreover, serotypes 1, 7, 10, 12, and 18 were widely detected in oral fluids, confirming their presence among Spanish herds, although they were rarely observed in acute pleuropneumonia outbreaks in this study. Consequently, we suggest that serotypes 1, 7, 10, 12, 18, and 19, despite being present, may pose a lower risk of causing serious illness in Spanish herds.

This fact is particularly unexpected for serotype 1, whose implicated virulence in clinical cases has been widely described in North America and Asia [42]. Its apparently lower virulence in Spain cannot be reliably confirmed due to the lack of data, which could influence the course and severity of the disease. Some of these data concern the bacterium itself, such as the detailed study of its Apx I toxin secretion system, while others involve the animals, namely, uncertainty regarding their vaccination status or the specific toxoid content of the vaccines used.

In the past 5 years, a heterogeneous epidemiology of pleuropneumonia has been described in Spain. This local study revealed significant disparities in the presence of serotypes between diseased and non-diseased animals. Serotypes 9/11, 2, 13, 17, 4, and 5 were the most frequently associated with clinical illness, however, nearly all serotypes could potentially possess sufficient virulence to cause illness under certain circumstances. The remaining known serotypes, with the exception of serotype 16 for which no evidence was found, were detected in oral fluid samples from apparently healthy animals.

This is the first report in which the *A. pleuropneumoniae* serotype is determined directly from the lungs via qPCR. This robust molecular method demonstrated that microbiological isolation can be avoided, allowing for a more rapid diagnosis of clinical cases. The direct detection can also be performed when monitoring samples such as oral fluids to reveal which serotypes circulate among the herd. The epidemiological data presented in this work can offer valuable criteria for the adoption of measures to control swine pleuropneumonia.

## Supplementary Information

**Additional file 1. Effect of pooling of lung samples on** ***A. pleuropneumoniae*** **and serotypes detection. Sensitivity assessment of pooling effect up to five animals within an outbreak.**

**Additional file 2. Validation results of** ***A. pleuropneumoniae*** **(APPL) and serotypes (AP01–AP19) qPCR kits.** Sensitivity and specifity assessment of qPCR assays used for detecting the *A. pleuropnemoniae* serotypes.

## Data Availability

The qPCR data used to support the findings of this study are available from the corresponding author upon reasonable request.
