# Correction: Serotype diversity of *Actinobacillus pleuropneumoniae* detected by real-time PCR in clinical and subclinical samples from Spanish pig farms during 2017–2022

**DOI:** 10.1186/s13567-025-01564-2

**Published:** 2025-06-20

**Authors:** José Luis Arnal Bernal, Marcelo Gottschalk, Sonia Lacotoure, Celia Sanz Tejero, Gema Chacón Pérez, Desiree Martín-Jurado, Ana Belén Fernández Ros

**Affiliations:** 1 Exopol. Pol, Río Gállego D14, San Mateo de Gállego, Zaragoza, Spain; 2 Research Group on Infectious Diseases in Production Animals and Swine and Poultry Infectious Diseases Research Centre, Faculty of Veterinary Medicine, University of Montreal, 3200 Sicotte, Saint-Hyacinthe, QC J2S 2M2 Canada

**Correction: Veterinary Research (2024) 55:165** 10.1186/s13567-024-01419-2

Following publication of the original article [1], the authors identified an inconsistency between the description of results in the main text and the data presented in Table 3. Specifically, the presence of Apx toxin genes does not align. The authors verified that the information in the table is accurate and should be considered correct.

The incorrect text is:

All serotype 2 biotype I isolates, except one, harboured ApxII coding genes exclusively (IBD, IICA). By contrast, all serotype 2 biotype II isolates also possessed ApxIII coding genes (IIICA and IIIBD).

The correct text is:

All serotype 2 biotype I isolates, except one, possessed ApxII (IBD, IICA) and ApxIII coding genes (IIICA, IIIBD). By contrast, all serotype 2 biotype II isolates harboured ApxII coding genes exclusively (IBD, IICA).
