# Supplementary material for: Serotype diversity of Actinobacillus pleuropneumoniae detected by real-time PCR in clinical and subclinical samples from Spanish pig farms during 2017–2022
Source: Vet Res. 2024 Dec 18;55:165. doi: 10.1186/s13567-024-01419-2 (PMC11654185; doi:10.1186/s13567-024-01419-2)
Supplement: Supplementary file 1 — Additional file 1. Effect of pooling of lung samples on A. pleuropneumoniae and serotypes detection. Sensitivity assessment of pooling effect up to five animals within an outbreak. [file 13567_2024_1419_MOESM1_ESM.docx]

**Additional file 1 Effect of pooling of lung samples on *A. pleuropneumoniae* and serotypes detection**

|  |  | | *A. pleuropneumoniae* qPCR (Cq value) | | Serotype qPCR  (Cq value) | |
| --- | --- | --- | --- | --- | --- | --- |
|  | Microbiological culture | Serotype | Single | Pool | Single | Pool |
| Lung01 | neg |  | neg |  |  |  |
| Lung02 | neg |  | neg |  |  |  |
| Lung03 | neg |  | neg |  |  |  |
| Lung04 | neg |  | neg |  |  |  |
| Lung05 | *A. pleuropneumoniae* | 13 | 30.5 | 33.8 | 27.5 | 30.3 |
| Lung06 | *A. pleuropneumoniae* | 17 | 24.3 | 26.6 | 22.5 | 26.7 |
| Lung07 | *A. pleuropneumoniae* | 2 | 28.1 | 30.6 | 28.0 | 30.0 |
| Lung08 | *A. pleuropneumoniae* | 4 | 26.1 | 28.1 | 26.1 | 28.1 |
| Lung09 | *A. pleuropneumoniae* | 8 | 27.0 | 28.2 | 27.1 | 28.7 |
| Lung10 | *A. pleuropneumoniae* | 9/11 | 24.2 | 26.2 | 23.6 | 26.0 |

Lungs from four pigs (01-04) with negative results for *A. pleuropneumoniae* qPCR and microbiological isolation were used to create pools of five lungs. Each positive lung (05-10) was mixed with the four negative lungs to form various pools of five tissue samples. The Cq values for *A. pleuropneumoniae* qPCR and the respective serotypes were compared between individual analyses and pooled samples. No sensitivity issues were reported for either *A. pleuropneumoniae* or the serotypes. This assessment supports the conclusion that pooling up to five animals within an outbreak does not compromise the sensitivity of the technique
