# Supplementary material for: Serotype diversity of Actinobacillus pleuropneumoniae detected by real-time PCR in clinical and subclinical samples from Spanish pig farms during 2017–2022
Source: Vet Res. 2024 Dec 18;55:165. doi: 10.1186/s13567-024-01419-2 (PMC11654185; doi:10.1186/s13567-024-01419-2)
Supplement: Supplementary file 2 — Additional file 2. Validation results of A. pleuropneumoniae (APPL) and serotypes (AP01–AP19) qPCR kits. Sensitivity and specifity assessment of qPCR assays used for detecting the A. pleuropnemoniae serotypes. [file 13567_2024_1419_MOESM2_ESM.docx]

**Additional file 2 Validation results of *A. pleuropneumoniae* (APPL) and serotypes (AP01-AP19) qPCR kits.**

|  |  | **EXOone APPL** | **EXOone**  **AP01-AP19** | | | | | | | | | | | | | | | | | |
| --- | --- | --- | --- | --- | --- | --- | --- | --- | --- | --- | --- | --- | --- | --- | --- | --- | --- | --- | --- | --- |
| **Strains** | **Serotype** |  | **1** | **2** | **3** | **4** | **5** | **6** | **7** | **8** | **9/11** | **10** | **12** | **13** | **14** | **15** | **16** | **17** | **18** | **19** |
| 4074 | 1 | 16.62 | 15.79 | - | - | - | - | - | - | - | - | - | - | - | - | - | - | - | - | - |
| 4226 | 2 | 15.45 | - | 15.78 | - | - | - | - | - | - | - | - | - | - | - | - | - | - | - | - |
| S1421 | 3 | 15.04 | - | - | 18.4 | - | - | - | - | - | - | - | - | - | - | - | - | - | - | - |
| M62 | 4 | 16.51 | - | - | - | 16.95 | - | - | - | - | - | - | - | - | - | - | - | - | - | - |
| K17 | 5 | 14.93 | - | - | - | - | 15.36 | - | - | - | - | - | - | - | - | - | - | - | - | - |
| FEM | 6 | 18.12 | - | - | - | - | - | 14.02 | - | - | - | - | - | - | - | - | - | - | - | - |
| WF83 | 7 | 14.3 | - | - | - | - | - | - | 14.09 | - | - | - | - | - | - | - | - | - | - | - |
| Minn 405 | 8 | 17.29 | - | - | - | - | - | - | - | 16.22 | - | - | - | - | - | - | - | - | - | - |
| CVJ13261 | 9 | 16.73 | - | - | - | - | - | - | - | - | 15.69 | - | - | - | - | - | - | - | - | - |
| 13039 | 10 | 17.27 | - | - | - | - | - | - | - | - | - | 15.89 | - | - | - | - | - | - | - | - |
| 56153 | 11 | 15.33 | - | - | - | - | - | - | - | - | 17.96 | - | - | - | - | - | - | - | - | - |
| 8328 | 12 | 15.99 | - | - | - | - | - | - | - | - | - | - | 16.45 | - | - | - | - | - | - | - |
| N-273 | 13 | 16.86 | - | - | - | - | - | - | - | - | - | - | - | 13.45 | - | - | - | - | - | - |
| 3906 | 14 | 17.05 | - | - | - | - | - | - | - | - | - | - | - | - | 16.78 | - | - | - | - | - |
| HS-143 | 15 | 14.91 | - | - | - | - | - | - | - | - | - | - | - | - | - | 16.32 | - | - | - | - |
| A-85 | 16 | 17.73 | - | - | - | - | - | - | - | - | - | - | - | - | - | - | 16.91 | - | - | - |
| 16287-1 | 17 | 14.53 | - | - | - | - | - | - | - | - | - | - | - | - | - | - | - | 14.37 | - | - |
| 7311555 | 18 | 14.75 | - | - | - | - | - | - | - | - | - | - | - | - | - | - | - | - | 14.96 | - |
| A8-013 | 19 | 15.99 | - | - | - | - | - | - | - | - | - | - | - | - | - | - | - | - | - | 12.96 |

The qPCR kits used in this study were validated using a collection of reference strains provided by the University of Montreal. The serotype of the reference strains was confirmed following the protocol described by Stringer et al. [17]. The *A. pleuropneumoniae* qPCR kit tested positive for all serotypes, while each serotyping qPCR assay tested positive only for the strain of the corresponding serotype. Positive results are represented by the respective Cq values, and negative results are indicated as “-”.
